# “How Much is that Player in the Window? The One with the Early Birthday?” Relative Age Influences the Value of the Best Soccer Players, but Not the Best Businesspeople

**DOI:** 10.3389/fpsyg.2016.00084

**Published:** 2016-02-02

**Authors:** Philip Furley, Daniel Memmert, Matthias Weigelt

**Affiliations:** ^1^Institute of Cognitive and Team/Racket Sport Research, German Sport UniversityKöln, Germany; ^2^Department Sport and Health, University of PaderbornPaderborn, Germany

**Keywords:** relative age effects, birthdate, Soccer, talent, development

Although people in general tend to attribute success to individual merit (see e.g., Gilbert and Malone, 1995) research has shown that something as trivial as the date of a person's birth can have—under certain circumstances—a major impact on an individual's achievement. The Canadian psychologist Roger Barnsley (Barnsley et al., 1985) made the extraordinary discovery that the great majority of top-level athletes were born within the first months of the year, whereas a lot less players playing at the highest level were born later in the year (Dudink, 1994; Edwards, 1994; Cobley et al., 2009). This skewed birthdate distribution was termed Relative Age Effect. The assumed explanation for this effect was simple and had nothing to do with Astrology, but instead was attributed to the fact that children and youth athletes are divided into age-groups according to their birth date (usually with the cutoff date being the first of January). This early selection cutoff date can lead to a maturation head start of almost a year within an age group. It is further assumed that this maturation head start will result in a Matthew effect (“the rich get richer; the poor get poorer,” see e.g., Merton, 1957) due to better developmental circumstances, such as better coaching, more playing and practice time. Indeed, research suggests that coaches have more favorable attitudes toward more matured players (Furley and Memmert, 2015) and that maturation advantages have the potential to translate to performance advantages (Buchheit and Mendez-Villanueva, 2014; Gastin and Bennett, 2014). As a consequence, the most successful athletes are likely to show a skewed birthdate distribution (see e.g., Helsen et al., 2012 for a recent demonstration), whereas success in other domains without early selection processes involving cutoff dates should not show a skewed birthdate distribution as individuals have equal opportunities to develop (Barnsley et al., 1985).

To date Relative Age Effects have almost exclusively been shown within high achieving cohorts, such as national teams or within the highest levels of competitive sport, while it remains unclear whether birthdates also have the potential to translate to monetary value. Therefore, we tested for Relative Age Effects within the 100 most valuable soccer players according to their 2015 CIES (Poli et al., 2015) estimated transfer values (high achievement group with early selection cutoff date) and within the 100 richest billionaires according to their *Forbes* Net Value (high achievement group without early selection cutoff date). See Supplemental Material and Author Note for more detail on the analyzed sample.

As expected, we found that amongst the 100 most valuable soccer players 60% were born in the first half of the year (*M*_value_ = 48.3 million $; *SD*_value_ = 41.1 million $), whereas 40% (*M*_value_ = 39.8 million $; *SD*_value_ = 15.1 million) were born in the second half, which differed significantly form the expected even distribution [$\chi_{\left(1,\;N=100\right)}^{2}=4.000$; *p* = 0.023, one-tailed; *OR* = 1.50 (0.86, 2.62); see Figure 1]. Although, players born in the first half of the year were worth approximately 8 Million $ more, this effect only approached significance [*t*_(80.380)_=1.472, *p* = 0.07, one-tailed; *d* = 0.30 (–0.10, 0.70)]. Importantly, no such skewed distribution was evident amongst the 100 highest ranked *Forbes* billionaires [$\chi_{\left(1,N=100\right)}^{2}=0.040$; *p* = 0.841, two-tailed; *OR* = 0.96 (0.55, 1.67)] with 49% being born in the first half year (*M*_netvalue_ = 22.0 billion$; *SD*_netvalue_ = 13.3 billion) and 51% (*M*_netvalue_ = 23.0 billion$; *SD*_netvalue_ = 14.3 billion) in the second half year. Comparative analysis of both the soccer and the *Forbes* distribution revealed a significant difference between the birthdate distributions of the 100 most valuable—in monetary terms—soccer players and businesspeople [$\chi_{\left(1,\;N=100\right)}^{2}=6.829$; *p* = 0.008, one-tailed; *OR* = 1.56 (0.89, 2.73)].

**Figure 1:**
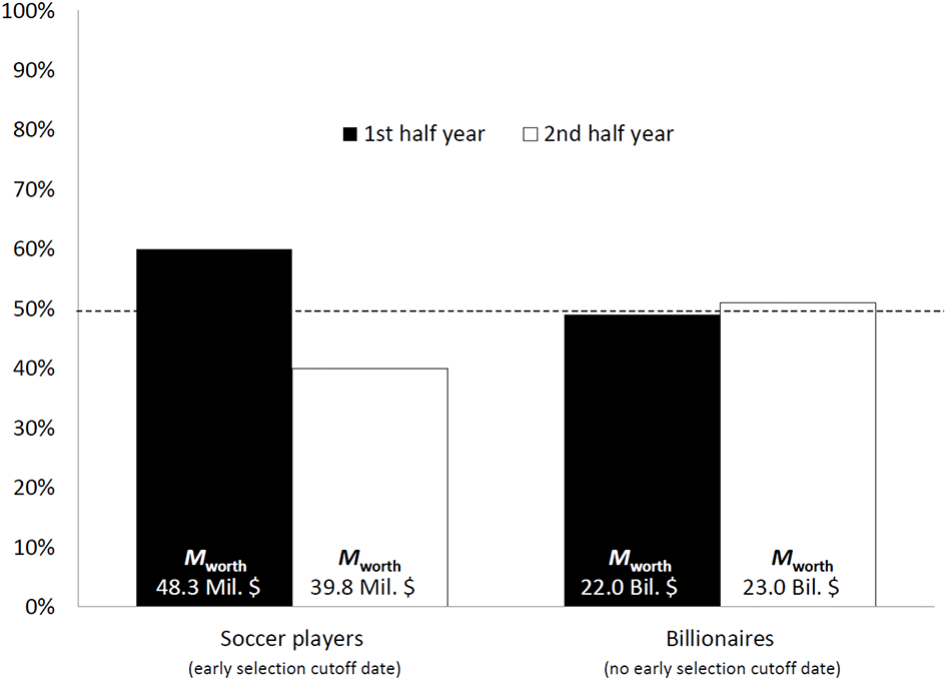
**Distributions of the 100 most valuable soccer players and billionaires as a function of half year in which they were born**. The dotted line represents the expected even distribution. The text within the bars shows the mean value of the respective groups.

The present analysis shows that birthdates—under certain circumstances (early selection cutoff dates)—not only have the potential to open up gates to high achieving cohorts, but can actually result in higher monetary value (but see Ashworth and Heyndels, 2007, for differences in actual earnings). While the division of children into age groups based on their birth-date has the well-meant intention to provide equal opportunities for participation and success, paradoxically this is not the case as there is a systematic exclusion of children born further away from the cutoff date (usually later in the year if the cutoff date is the 1st of January). Here, it is important to note that Relative Age Effects have also been found in the broader educational system (Pidgeon, 1965), albeit the pattern is not very consistent across studies (e.g., Jeronimus et al., 2015 for a recent investigation). Of further relevance, Matsubayashi and Ueda (2015) argued that relative younger students seem to take less desirable career paths that might be associated with poorer psychological health compared to students with a relative age advantage. In this respect, we hope to add a further line of argumentation against highly-selective developmental and education systems beginning at early ages based on birthdates as people in domains that arguably are not affected (e.g., business) by early selection cutoff dates have the potential of doing equally well.

Taken together, early selection cutoff dates have broad implications that need to be taken seriously by political decision makers in order to meet the goal of providing equal opportunities to people.

## Author note

As the estimated transfer values for the soccer players were published as ranges, we calculated the mean of the range for the conducted analyses. Two birthdates could not be obtained from the top 100 *Forbes* list (rank 32 and 52). Further, ranks 58, 69, and 80 were siblings with diverging birthdates and were therefore not included in the analyses. In order to have an equal sample size to the soccer players, we filled up the list with the subsequent ranks until rank 105.

## Author contributions

PF, DM, MW developed the study concept, and all authors contributed to the design and collected the data. PF analyzed it in collaboration with DM. PF wrote the first draft of the manuscript, and DM and MW helped edit and revise it. All authors approved the final, submitted version of the manuscript.

### Conflict of interest statement

The authors declare that the research was conducted in the absence of any commercial or financial relationships that could be construed as a potential conflict of interest.
